# Supplementary material for: Diagnostic serology test comparison for Q fever and Rift Valley fever in humans and livestock from pastoral communities
Source: PLoS Negl Trop Dis. 2024 Oct 14;18(10):e0012300. doi: 10.1371/journal.pntd.0012300 (PMC11501034; doi:10.1371/journal.pntd.0012300)
Supplement: S1 Table — (DOCX) [file pntd.0012300.s001.docx]

Supporting Information

**S1A Table: ID Screen Q Fever Indirect Multi-species kit**

**The wells are sensitised with Coxiella burnetii phase I and II antigens, isolated from bovine abortion placenta. Specific antibodies to Coxiella burnetti phase I and II, if present in the test samples, form an antigen-antibody complex. After washing, an anti-multi-species conjugate is distributed to the wells. It binds to the antibodies, forming a complex. The reaction is revealed by a reagent solution. The resulting staining is related to the amount of specific antibody present in the sample: A) In the presence of antibodies in the sample, coloration appears. B) In the absence of antibodies in the sample, there is no coloration. The OD was recorded at 450 nm. For each sample, the percentage S/P (S/P %) was calculated.**

| Result | Status |
| --- | --- |
| S/P % ≤ 40% | negative |
| 40% < S/P % ≤ 50% | Equivocal |
| 50% < S/P % ≤ 80% | positive |
| S/P % > 80% | Strongly positive |

**S1B Table: Panbio Coxiella Burnetii (Q Fever) IgG ELISA**

**If the sample contains antibodies against the C. burnetii antigen, these antibodies bind to the C. burnetii antigen, which adheres to the surface of the microwells. Serum residues are removed by washing, and anti-human IgG is added. The microwells are washed, and a chromogen is added. The chromogen is hydrolyzed by the enzyme and turns blue at first and yellow after the reaction is stopped. The color development is an indicator for the presence of C. burnetii IgG antibodies in the test sample. An index value can be calculated by dividing the sample absorbance by the cut-off value.**

| INDEX | Status | Interpretation |
| --- | --- | --- |
| <0.9 | Negative | No detectable IgG antibodies. |
| 0.9 – 1.1 | Equivocal | Equivocal samples should be repeated. |
| >1.1 | Positive | Presence of detectable IgG antibodies suggests recent or past exposure. |

**S1C Table: ID Screen Rift Valley Fever Competition Multi-species kit**

**This diagnostic kit is designed to detect antibodies to the RVFV nucleoprotein (NP) in serum or plasma. Anti-nucleoprotein antibodies, if present, form an antigen-antibody complex that masks the epitopes of the nucleoprotein. An anti-nucleoprotein conjugate is distributed in the cups. It binds to the free epitopes of the nucleoprotein. The resulting coloration depends on the amount of specific antibodies present in the test sample. A) In the absence of antibodies, a coloration appears. B) In the presence of antibodies, no coloration appears. The OD was recorded at 450 nm. For each sample, the percentage of competition (S/N%) was calculated. OD(sample) / OD(negative control) x 100**

| Result | Status |
| --- | --- |
| S/N % ≤ 40 % | positive |
| 40 % < S/N % ≤ 50% | equivocal |
| S/N % > 50% | negative |

**S1D Table: IDEXX Q Fever Ab test (for ruminants)**

**Microtiter plates are supplied precoated with inactivated antigen. Dilutions of the samples to be tested are incubated in the wells of these plates. Any antibody directed against Coxiella burnetii binds to the antigen in the wells and forms an antigen/antibody complex on the plate well surface. Unbound material is removed from the wells by washing. A peroxidase labeled anti-ruminant IgG conjugate is added, which binds to the ruminant antibodies complexed with the Coxiella burnetii antigen. Unbound conjugate is removed by washing and the substrate is added to the wells. The degree of color that develops is directly proportional to the amount of antibody specific for Coxiella burnetii present in the sample. The result is obtained by comparing the optical density (OD) of the sample well with the OD from the positive control.**

| Result | Status |
| --- | --- |
| S/P % < 30% | negative |
| 30% < S/P % < 40% | suspect |
| S/P % ≥ 40% | positive |

**S1E Table: Q Fever IFA IgG assay (Focus Diagnostics, US)**

**Virulent isolates are of the phase I type, while serial passage in eggs or tissue culture is required for selection of the avirulent phase II transition. These phases are serologically distinguishable and useful in the serodiagnosis of both acute and chronic C. burnetii infections. The presence of IgG to phase II antigen is indicative of acute disease. The presence of IgG to phase I antigen is indicative of chronic disease. The IFA assay is a 2-stage “sandwich” procedure. In the first stage, the patient sera is diluted and added to the wells; each well contains 2 individual spots: a C. burnetii phase I antigen spot and a C. burnetii phase II antigen spot. Following incubation, the slide is washed in unbound serum antibodies are removed. In the second stage, each antigen well is overlaid with fluorescein-labeled antibody to IgG. The slide is incubated allowing antigen-antibody complexes to react with the fluorescein-labeled anti-IgG. After the slide is washed, dried, and mounted, it is examined using fluorescence microscopy. Positive reactions appear as bright apple-green fluorescent rickettsia with a background matrix of yolk sac. Semi-quantitative endpoint titers are obtained by testing serial dilutions of positive specimens.**

| IgG endpoint titer | Interpretation |
| --- | --- |
| ≥ 1:16 to both phase I  and phase II antigens | Positive. Strongly suggests C. burnetii infection. Phase I antibody titers of greater than or equal to phase II antibody titers are consistent with chronic infection or convalescent phase of Q fever. |
| < 1:16 to both phase I  and phase II antigens | Negative. No antibody detected. Argues against C. burnetii infection. This result is seen in persons with either no previous C. burnetii infection or with early infection. If early acute Q fever is suspected, perform IgM testing and obtain a serum second sample 2 to 3 weeks later and test in parallel with the original sample |

**S1F Table: Anti-Rift-Tal-Fieber-Viren-IIFT [IgG] from EUROIMMUN**

**In the first incubation step, specific antibodies from the diluted patient sample bind to the solid-phase bound antigens. In the next step, a fluorescein (FITC)-labeled antibody (conjugate) binds to the specific antibodies from the patient sample. By excitation with the respective wavelength, the complex can be made visible at the fluorescence microscope.**

| Anti-rift valley fever virus | Status |
| --- | --- |
| No reaction at 1:100 | Negative. No antibodies against RVF virus detectable in patient serum. |
| Positive reaction at 1:100 | Positive. |
