# Supplementary material for: Diagnostic serology test comparison for Q fever and Rift Valley fever in humans and livestock from pastoral communities
Source: PLoS Negl Trop Dis. 2024 Oct 14;18(10):e0012300. doi: 10.1371/journal.pntd.0012300 (PMC11501034; doi:10.1371/journal.pntd.0012300)
Supplement: S2 Table — (DOCX) [file pntd.0012300.s002.docx]

**S2 Table: Sample characteristics of a random sample from the study population in Özcelik et al., 2023**

|  | Human samples |  | Livestock samples |  |
| --- | --- | --- | --- | --- |
|  | **n** | **%** | **n** | **%** |
| Total | 91 | 100 | 102 | 100 |
|  |  |  |  |  |
| District |  |  |  |  |
| Danamadji | 56 | 62 | 58 | 57 |
| Yao | 35 | 38 | 44 | 43 |
|  |  |  |  |  |
| Setting |  |  |  |  |
| Village | 40 | 44 | 43 | 42 |
| Camp | 51 | 56 | 59 | 58 |
|  |  |  |  |  |
| Sex |  |  |  |  |
| Male | 65 | 71 | 31 | 30 |
| Female | 26 | 29 | 71 | 70 |
|  |  |  |  |  |
| Age |  |  |  |  |
| Age group 1 | 27 | 30 | 17 | 17 |
| Age group 2 | 26 | 28 | 51 | 50 |
| Age group 3 | 30 | 33 | 34 | 33 |
| Age group 4 | 8 | 9 | – | – |
|  |  |  |  |  |
| Species |  |  |  |  |
| Cattle | – | – | 47 | 46 |
| Small ruminants | – | – | 42 | 41 |
| Equids | – | – | 13 | 13 |
