# Supplementary material for: Diagnostic serology test comparison for Q fever and Rift Valley fever in humans and livestock from pastoral communities
Source: PLoS Negl Trop Dis. 2024 Oct 14;18(10):e0012300. doi: 10.1371/journal.pntd.0012300 (PMC11501034; doi:10.1371/journal.pntd.0012300)
Supplement: S3 Table — (DOCX) [file pntd.0012300.s003.docx]

**S3 Table: Results of univariable logistic regression models investigating the effect of demographic factors as independent variables on the inter-laboratory test agreement of Q fever (QF) and Rift Valley Fever (RVF) in humans and livestock. Goodness of fit of the models are presented as pseudo-R-squared (R2). Bold p-values indicate significance based on a threshold of 0.05.**

|  | QF livestock | QF humans | RVF livestock | RVF humans |
| --- | --- | --- | --- | --- |
| Covariates (OR and CI)  R^2^ | | | | |
| District  (Reference: Danamadji) | Yao:  0.29 (0.05, 1.56)  P = 0.15  R^2^ = 0.06 | Yao:  1.49 (0.56, 3.95)  P = 0.43  R^2^ = 0.01 | Yao:  1.62 (0.28, 9.25)  P = 0.59  R^2^ = 0.008 | Yao:  1.14 (0.38, 3.43)  P = 0.82  R^2^ = 0.001 |
| Setting  (Reference: camp) | Village:  0.25 (0.05, 1.41)  P = 0.12  R^2^ = 0.07 | Village:  6.34 (2.13, 18.93)  **P = 0.0009**  R^2^ = 0.23 | Village:  0.68 (0.13, 3.54)  P = 0.65  R^2^ = 0.006 | Village:  1.51 (0.51, 4.53)  P = 0.46  R^2^ = 0.01 |
| Species  (Reference: cattle) | Equids:  1.37 (0.14, 13.5)  P = 0.79  Small ruminants:  2.11 (0.36, 12.3)  P = 0.40  R^2^ = 0.17 | - | Equids:  Inf (perfect agreement in equids)  P = 0.81  Small ruminants:  0.93 (0.18, 4.88)  P = 0.93  R^2^ = 0.13 | - |
| Age | Per year:  0.87 (0.66, 1.15)  P = 0.32  R^2^ = 0.02 | Per 10 years:  0.94 (0.69, 1.28)  P = 0.69  R^2^ = 0.003 | Per year:  0.87 (0.66, 1.15)  P = 0.32  R^2^ = 0.02 | Per 10 years:  0.8 (0.58, 1.1)  P = 0.17  R^2^ = 0.03 |
| Age group (Reference: age group 1/group 2*) | Group 2:  1.57 (0.13, 18.66)  P = 0.72  Group 3:  0.46 (0.05, 4.56)  P = 0.51  R^2^ = 0.05 | Group 2:  0.95 (0.24, 3.75)  P = 0.94  Group 3:  0.53 (0.14, 1.96)  P = 0.34  Group 4:  0.31 (0.05, 1.94)  P = 0.21  R^2^ = 0.05 | Group 1*:  Inf (perfect agreement)  P = 0.72  Group 3*:  0.64 (0.12, 3.37)  P = 0.60  R^2^ = 0.07 | Group 2:  0.69 (0.14, 3.42)  P = 0.65  Group 3:  0.39 (0.09, 1.71)  P = 0.21  Group 4:  0.21 (0.03, 1.35)  P = 0.10  R^2^ = 0.06 |
| Sex  (Reference: male) | Female:  0.35 (0.04, 3.07)  P = 0.34  R^2^ = 0.03 | Female:  0.82 (0.27, 2.48)  P = 0.72  R^2^ = 0.002 | Female:  0 (perfect agreement in males)  P = 0.23  R^2^ = 0.12 | Female:  0.97 (0.30, 3.09)  P = 0.96  R^2^ = 0.00005 |
