# Supplementary material for: Diagnostic serology test comparison for Q fever and Rift Valley fever in humans and livestock from pastoral communities
Source: PLoS Negl Trop Dis. 2024 Oct 14;18(10):e0012300. doi: 10.1371/journal.pntd.0012300 (PMC11501034; doi:10.1371/journal.pntd.0012300)
Supplement: Attachment — Submitted filename: PNTD-D-24-00848_Review.docx [file pntd.0012300.s007.docx]

**Diagnostic serology test comparison for Q fever and Rift Valley fever in humans and livestock from pastoral communities**

In the manuscript, Hungerbühler et al. report the "**Diagnostic serology test comparison for Q fever and Rift Valley fever in humans and livestock from pastoral communities**". I want to congratulate the authors on a relatively well-designed study and a well-written manuscript. This study makes a significant contribution to our understanding of the diagnosis of vector-borne infectious diseases. Below, I have outlined some minor points that need to be addressed.

**Minor concerns:**

Line 57: could the authors state what the routine test is? I assume it’s ELISA.

Line 71: please put a reference.

Line 74: put "clinical" between severe and manifestations.

Line 85 – 87: The virus neutralization test (VNT) is not only the most specific, but also considered the gold standard for most vector-borne infections. However, authors could also comment on VNT's limitations including turnaround time and the variability in results mainly related to cell and virus passage, virus strain, etc. Also, biosecurity requirements being a hindrance especially in developing countries.

Line 127. This study was approved …. I suggest you delete has been submitted to and.

Line 222: male instead on men.

Line 277 – 279: The authors report a higher level of inter-laboratory agreement for livestock QF than they had anticipated. It would be helpful for them to revise the wording to avoid implying preconceived assumptions. Additionally, it would be beneficial for them (and the readers) to discuss the potential reasons for this higher level in comparison to the levels reported by others.

Line 292 – 294: Could the authors briefly discuss the key differences between ELISA and IFA as the cause of differences observed? Which one is more sensitive, specific, prone to human errors, and affected by buffers, etc.

Line 296 – 98: could the authors confirm if the samples sent to Germany and Switzerland were aliquots from the original samples collected in Chad. If so, could the authors discuss why haemolysis affected one test but not the other? Maybe assay formats, reagents/buffers used, etc. Also, could you clarify whether the labs in Germany and/or Switzerland received serum/plasma or whole blood? This could be used to discuss the haemolysis effect in tests performed in Europe as to those performed in Chad.

Line 307 – 308: I agree that repeated freeze-thaw cycles may affect the test outcome, especially the predominant IgM during the early immune response, which would break down in such circumstances.

Line 313 – 3022: authors may also discuss the effects of immune maturation, in addition to the repetitive exposure and cross-reactivity in older individuals.

Line 318: change provoked by another term. Maybe consider using "induced".

Line 322: add space between references and the word in.

Line 345: larger range or wider range?

Line 352: what do authors mean by statistically robust? It is a bit confusing, please clarify.

Line 352 – 356: could the authors explain the connection between the inflammatory response and the humoral immune response? Are the authors implying that the innate immune response, such as inflammation, negatively impacts the humoral immune response? This aspect is ambiguous and may not be entirely accurate. It would be helpful if the authors could provide further explanation.

**General comment for conclusion:** Authors should also discuss and emphasize the use of gold standard tests to accurately report the prevalence of vector borne infections disease. In many cases, ELISA is used for initial screening, while VNTs should be conducted on ELISA positive samples for confirmation.
