# Supplementary material for: Diagnostic serology test comparison for Q fever and Rift Valley fever in humans and livestock from pastoral communities
Source: PLoS Negl Trop Dis. 2024 Oct 14;18(10):e0012300. doi: 10.1371/journal.pntd.0012300 (PMC11501034; doi:10.1371/journal.pntd.0012300)
Supplement: Attachment — Submitted filename: response to reviewers.docx [file pntd.0012300.s008.docx]

Liebefeld, 28.08.2024

Valerie Hungerbühler

valerie.hungerbuehler@unibe.ch

### VPH Institute, Schwarzenburgstr. 155, CH-3097 Liebefeld

valerie.hungerbuehler@unibe.ch

www.vphi.ch

Dr. med. vet. Valerie Hungerbühler

VPH Institute

Vetsuisse Faculty, University of Bern

Schwarzenburgstrasse 155

CH-3097 Liebefeld, Switzerland

Vetsuisse Faculty, University of Bern

Dept. Clinical Research & VPH

VPH Institute

Re: Response to the Reviewers

Dear Dr Mshelbwala, Dr Viennet, and reviewers

Thank you for reviewing my revised manuscript titled "Diagnostic serology test comparison for Q fever and Rift Valley fever in humans and livestock from pastoral communities." I appreciate the valuable feedback and constructive comments you provided!

In the following, I have carefully addressed each reviewer's comments, and the changes made are highlighted using “track changes” in the revised manuscript.

Kind regards,

Valerie Hungerbühler

**Reviewer #1:** This study sought to address an often-overlooked concern with seroprevalence studies. The sampling technique used, the diagnostic performance of the laboratory test, the diagnostic quality of the sample type, and the lab-to-lab variation in out-of-kit reagents, personnel, and SOPs are important factors that drive the validity of findings from such studies. Specifically, this study evaluates the agreement of Q fever and RVF serological results in a laboratory in Chad compared to results from laboratories in 2 European countries. The findings of this study are most relevant to epidemiologists.

Overall, the authors were clearly able to justify the study and discuss the results in-depth. I have a few concerns:

**Response: Thank you very much for taking the time to review our manuscript and for providing such detailed and constructive feedback. We have implemented your suggestions accordingly. Below, you will find a detailed list of your comments and our corresponding responses, including line numbers where the changes can be found in the manuscript with tracked changes.**

**Comment:** Line 138: I missed the portion of the methods that explains how they came about the sample size; it would be great if the authors included some sample size justification, if there was one, or indicate if it was non-probabilistic.

**Response:** I have made the following changes to the manuscript: The samples analyzed in this study were collected between January and February 2018, as reported by Özcelik, R., et al. I have added the highlighted part to the sentence in line 140. Accordingly, the sampling procedure for the 571 and 389 human samples, as well as the 560 and 483 livestock samples (from Danamadji and Yao, respectively), is thoroughly detailed by Özcelik, R., et al. (DOI: 10.1371/journal.pntd.0011395). For clarification, I also summarized the method for sample size determination (lines 143 – 147).

The decision to use a 10% subsample of the original sample size [4] was based on the resources available. I have added this information in line 162/163.

**Comment:** Lines 140 to 161: In studies to investigate the effect of a variable on an outcome, it is best practice to keep every other variable constant (as much as is within the power of the investigator). The authors used only the Q fever Indirect ELISA by IDvet for livestock sample testing in Chad but used the same test only for equids in Switzerland. A different kit was used for ruminants. The authors were silent about this noteworthy discrepancy in their comparisons and discussions. Would the authors mind sharing why this does not influence their findings? Considering the study's design aimed to investigate the lab-to-lab or place-to-place differences in test results, is there a reason why the same tests were not used in the laboratories (also the RVF testing for Humans that used a different test in Europe)?

**Response:** I have made the following changes to the manuscript: The objective of this study was to assess the inter-laboratory agreement of standard diagnostic tests utilized by the participating laboratories, measured by the concordance, Cohen's kappa, and PABAK. I have added the highlighted part to the sentence in line 120/121.

Therefore, the aim of our study was not to examine reproducibility using the same test, but to observe the "real-world" agreement by selecting tests typically used in routine diagnostics. For Q fever diagnostics in ruminants, an ELISA specifically adapted for automation in ruminants was utilized in Switzerland. However, an equivalent test was not available on the Swiss market for horses. Consequently, the same ELISA used for horse samples in Chad was applied.

I have also added a section to discuss how this could have influenced the finding: lines 332 – 338.

**Comment:** Lines 138, 148, 203, 206, 209, 218: It is unclear how the 10% mentioned in line 148 matches the sample numbers in 138. The final number of samples mentioned in lines 206 and 218 does not match the numbers in the last row of Table 1. Would the authors be implying that the total number of livestock samples used was 102 because 1 sample that was tested in the Q fever group could not be tested for RVF? If that is the message being conveyed, the authors may want to adjust line 206 to read, “91 human and 102 livestock samples were tested for either one OR both tests.” More text may be added in Figure 1 and other portions of the manuscript to make this clearer for the readers.

**Response:** Thank you for your valuable suggestion. I have adjusted line 194 as you recommended. Additionally, I have added more text to the caption of Figure 1 and revised the paragraph about the sample processing (lines 188 – 195).

**Comment:** Line 197: the function exp(coefficients(model)) does not give an upper CI. Please revise lines 197 and 198

**Response:** Thank you for this careful correction. I have made the adjustment in lines 236 – 239.

**Comment:** Lines 116-119: Please consider rewording your objective to be easier understood or piecing the objective into more than one sentence.

**Response:** I revised the objective accordingly (lines 120 – 124).

**Comment:** Figure 1: The authors may consider adding more details to the figure and title to let the readers grasp the full scope of what is being described without referring to the main manuscript text.

**Response:** I edited the figure accordingly.

**Reviewer #2:**

**Comment:** Minor editorial modifications required have been highlighted in the reviewed manuscript attached to this submission.

**Response: Thank you very much for your thorough review. I have carefully considered each of your comments and corrections and edited the manuscript accordingly.**

**Comment:** The authors should include the details of sample size determination. This information is crucial for research reproducibility.

**Response:** Thank you for your comment. I have made changes to the manuscript in lines 139 to 150 and 162/163 to address this concern.

**Reviewer #3:** In the manuscript, Hungerbühler et al. report the "**Diagnostic serology test comparison for Q fever and Rift Valley fever in humans and livestock from pastoral communities**". I want to congratulate the authors on a relatively well-designed study and a well-written manuscript. This study makes a significant contribution to our understanding of the diagnosis of vector-borne infectious diseases. Below, I have outlined some minor points that need to be addressed.

**Response: Thank you for thoroughly reviewing our manuscript and for offering such insightful and constructive feedback. We have carefully considered your suggestions and made the necessary revisions. Below, you will find a detailed list of your comments along with our responses, including the line numbers where the changes have been made in the manuscript with tracked changes.**

**Comment:** Line 57: could the authors state what the routine test is? I assume it’s ELISA.

**Response:** I have made the changes to the manuscript accordingly (line 58).

**Comment:** Line 71: please put a reference.

**Response:** I have made the changes to the manuscript accordingly (line 73).

**Comment:** Line 74: put "clinical" between severe and manifestations.

**Response:** I have made the changes to the manuscript accordingly (line 75).

**Comment:** Line 85 – 87: The virus neutralization test (VNT) is not only the most specific, but also considered the gold standard for most vector-borne infections. However, authors could also comment on VNT's limitations including turnaround time and the variability in results mainly related to cell and virus passage, virus strain, etc. Also, biosecurity requirements being a hindrance especially in developing countries.

**Response:** I have included information about VNT’s limitations in the section from lines 86 to 90.

**Comment:** Line 127. This study was approved …. I suggest you delete has been submitted to and.

**Response:** I have made the changes to the manuscript accordingly (line 132).

**Comment:** Line 222: male instead on men.

**Response:** Thank you very much for this careful correction. I have made the changes to the manuscript accordingly (line 263).

**Comment:** Line 277 – 279: The authors report a higher level of inter-laboratory agreement for livestock QF than they had anticipated. It would be helpful for them to revise the wording to avoid implying preconceived assumptions. Additionally, it would be beneficial for them (and the readers) to discuss the potential reasons for this higher level in comparison to the levels reported by others.

**Response:** I have revised this section (lines 326 to 332) and included a discussion on the potential reasons for the differences in agreement levels compared to those reported by others.

**Comment:** Line 292 – 294: Could the authors briefly discuss the key differences between ELISA and IFA as the cause of differences observed? Which one is more sensitive, specific, prone to human errors, and affected by buffers, etc.

**Response:** I have revised this section (lines 357 to 363), adding information about the ELISA and IFA used in the study, as well as the methodological differences that could have led to inter-laboratory disagreement.

**Comment:** Line 296 – 98: could the authors confirm if the samples sent to Germany and Switzerland were aliquots from the original samples collected in Chad. If so, could the authors discuss why haemolysis affected one test but not the other? Maybe assay formats, reagents/buffers used, etc. Also, could you clarify whether the labs in Germany and/or Switzerland received serum/plasma or whole blood? This could be used to discuss the haemolysis effect in tests performed in Europe as to those performed in Chad.

**Response:** The samples sent to Germany and Switzerland were aliquots, and the laboratories received serum. I have clarified this by revising lines 38, 58, 189 – 191, Figure 1 and Table 1. Additionally, I have added a section discussing the potential consequences of hemolysis on the serum (lines 364 – 374).

**Comment:** Line 307 – 308: I agree that repeated freeze-thaw cycles may affect the test outcome, especially the predominant IgM during the early immune response, which would break down in such circumstances.

**Response:** All of the tests in this study detected IgG. I have clarified this by revising Figure 1. Additionally, I have added a section to the discussion (lines 382 – 387).

**Comment:** Line 313 – 3022: authors may also discuss the effects of immune maturation, in addition to the repetitive exposure and cross-reactivity in older individuals.

**Response:** I have added a discussion point on the effect of immunosenescence, as it relates to immune maturation, and I have found additional literature on its impact on antibody response. (lines 405 - 410).

**Comment:** Line 318: change provoked by another term. Maybe consider using "induced".

**Response:** I changed the manuscript accordingly.

**Comment:** Line 322: add space between references and the word in.

**Response:** I changed the manuscript accordingly.

**Comment:** Line 345: larger range or wider range?

**Response:** I changed the manuscript accordingly.

**Comment:** Line 352: what do authors mean by statistically robust? It is a bit confusing, please clarify.

**Response:** I have reformulated the sentence (lines 436 – 438).

**Comment:** Line 352 – 356: could the authors explain the connection between the inflammatory response and the humoral immune response? Are the authors implying that the innate immune response, such as inflammation, negatively impacts the humoral immune response? This aspect is ambiguous and may not be entirely accurate. It would be helpful if the authors could provide further explanation.

**Response:** Due to the high p-values and wide confidence intervals of the OR, which suggest that the results lack strong evidence and may be influenced by variability or sample size issues, we decided to remove this part of the discussion.

**General comment for conclusion:** Authors should also discuss and emphasize the use of gold standard tests to accurately report the prevalence of vector borne infections disease. In many cases, ELISA is used for initial screening, while VNTs should be conducted on ELISA positive samples for confirmation.

**Response:** I have added a sentence to the conclusion (lines 453 – 454). Since the WHO/WOAH do not specify VNT as the gold standard for RVF or QF, I kept the wording more general.

**Reviewer #4:** The paper aims to investigate the differences between diagnostic tests of Q fever and RVF in livestock and human in different labs. The study design and the statistical analysis would support the testing of the comparison. However, the sampling design was not clearly introduced. The main concerns are:

**Response: We sincerely appreciate your thoughtful review of our manuscript and the valuable feedback you provided. We have addressed your suggestions and made the appropriate revisions. Please find below a detailed list of your comments, along with our responses, including the line numbers where the changes have been incorporated in the manuscript with tracked changes.**

**Comment:** More demographic information of livestock from the two areas is needed: by species: age, sex, herd sizes, and all livestock from one herd?

**Response:** Demographic information (species, age, sex) about the livestock is available in Table S7 (supplementary material). Regarding the number of herds, I have added a sentence (line 267/268). We did not include the herd size in our data collection.

**Comment:** for the clustering sampling: within each cluster how to choose the apparently healthy ones? how did the authors determine the sample size then?

**Response:** I have revised the manuscript for clarification: lines 143 – 150.

**Comment:** lines 202-207: this paragraph should be included in the method section

**Response:** I changed the manuscript accordingly.

**Comment:** Fig 1: Please add the testing methods, institutes and countries aside the buildings so easier for readers to understand

**Response:** I have revised Figure 1 accordingly.

**Comment:** line 222: suggest revising "men" to "males" as the studying group of <30 may had included boys

**Response:** Thank you for this careful correction. I have made the changes to the manuscript accordingly (line 263).

**Comment:** Table 3: the table looks incomplete at the bottom? and the "*" needs to be explained in a note under.

**Response:** I have made the changes to the manuscript accordingly (line 280).

In general, the conclusions were well presented. However, the limitation of the study could have been addressed better by discussing why not using the same methods in different labs to compare.

**Comment:** For example, authors mentioned the poor consistent may be due to different tests used in different labs, but why can't use the same IFA in Chad to compare? Need to discuss the challenge for having other tests than ELISA in Chad.

**Response:** I have made the following revisions to the manuscript: The objective of this study was to assess the inter-laboratory agreement of standard diagnostic tests employed by the participating laboratories. To clarify this, I have added the highlighted section to the objective in lines 120 - 124.

The aim of our study was not to assess reproducibility using the same test, but rather to evaluate "real-world" agreement by selecting tests typically used in routine diagnostics. Consequently, we did not discuss the use of different diagnostic tests as a limitation of this study.

Other comments:

**Comment:** Lines 270-273: No need to repeat this as it has been introduced.

**Response:** I have deleted this section as per your suggestion.

**Comment:** Line 291: please elaborate on the possible misinterpretations

**Response:** I have reviewed the references and revised the manuscript accordingly (lines 350 – 355).

**Comment:** Line 321: Since you mentioned the age had impacts on antibody titres, please advise on the implementation of this finding. For example, what if you adjust the cut-offs of reading results?

**Response:** I have incorporated your suggestion and revised the manuscript accordingly, as reflected in lines 411–413.

I believe the revisions have strengthened the manuscript, and I appreciate the opportunity to make these changes. I hope they adequately address your concerns.

Thank you once again for the opportunity to contribute to PLOS Neglected Tropical Diseases. I look forward to hearing from you soon.

Sincerely,

Valerie Hungerbühler, Dr. med. vet.

Residentin ECVPH

Veterinary Public Health Institut

Vetsuisse Fakultät Universität Bern

Schwarzenburgstr. 161

CH-3003 Bern-Liebefeld
